# Supplementary material for: Causal interplay between lactose intolerance and gut microbiota: a combined bidirectional Mendelian randomization and in vivo validation study
Source: Front Nutr. 2026 Jun 1;13:1803337. doi: 10.3389/fnut.2026.1803337 (PMC13265576; doi:10.3389/fnut.2026.1803337)
Supplement: Supplementary file 1 [file Data_Sheet_1.zip › supplementary materials/Forward/leave one out cross validation/ebi-a-GCST90027701.finngen_R12_E4_LACTONAS.pdf]

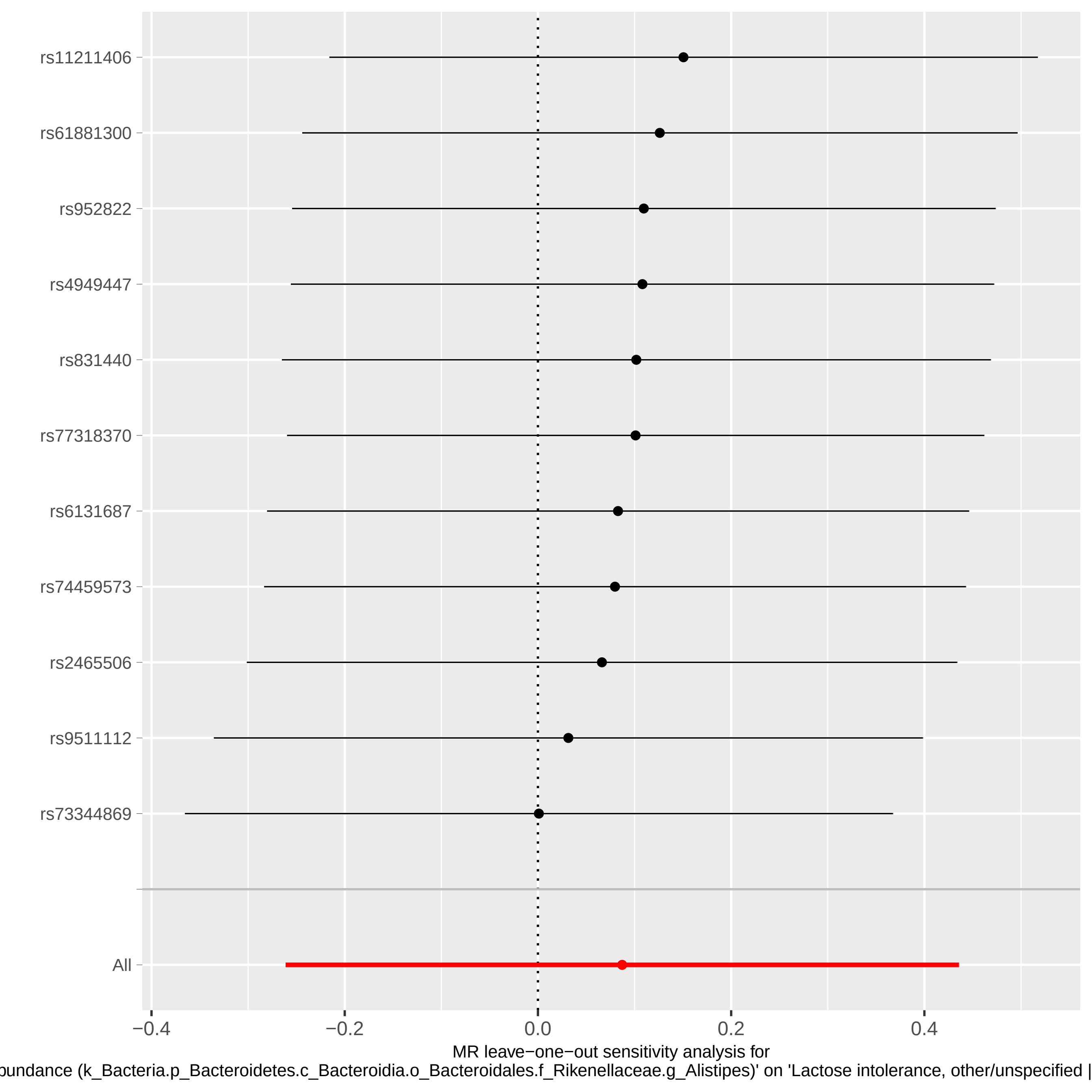
rs11211406
rs61881300
rs952822
rs4949447
rs831440
rs77318370
rs6131687
rs74459573
rs2465506
rs9511112
rs73344869
All
−0.4
−0.2
0.0
0.2
0.4
MR leave−one−out sensitivity analysis for
oundance (k_Bacteria.p_Bacteroidetes.c_Bacteroidia.o_Bacteroidales.f_Rikenellaceae.g_Alistipes)' on 'Lactose intolerance, other/unspecified |
